# Accuracy of Static Computer-Assisted Implant Placement in Narrow Ridge by Novice Clinicians

**DOI:** 10.1055/s-0045-1802949

**Published:** 2025-03-12

**Authors:** Jaafar Abduo, Radhwan Himmadi Hasan, Douglas Lau

**Affiliations:** 1 Melbourne Dental School, Melbourne University, Melbourne, Victoria, Australia; 2 Prosthodontics Department, College of Dentistry, University of Mosul, Mosul, Iraq

**Keywords:** computer-assisted, guided surgery, implant surgery, surgical guide

## Abstract

**Objective:**

This study aimed to evaluate the accuracy and clinical impact of implant placement by novice implant clinicians in the narrow anterior ridge by fully guided (FG), pilot-guided (PG), and freehand (FH) placements.

**Materials and Methods:**

A maxillary surgical model with missing incisors and a narrow ridge was designed. Two implants were planned in the lateral incisor locations to receive screw-retained implant prosthesis. Fifteen novice implant clinicians placed implants according to every placement. Angle, vertical and horizontal platform, and horizontal apex deviations from the planned implant were measured. The clinical impact evaluation aimed to relate the position of each placed implant to (1) periimplant bone dimension after implant placement and (2) the prosthesis retention mechanism.

**Results:**

The FG implants were more accurate than PG implants at the angle ( *p* = 0.001) and maximum horizontal apex deviations ( *p* = 0.001), and were more accurate than FH implants for all comparisons ( *p* = 0.001). The PG implants were superior to FH implants at the maximal horizontal platform deviation ( *p* = 0.001). All FG implants were fully covered with bone and could be restored with screw-retained prostheses. One PG implant (3.3%) had fenestration at the apex, and one PG implant (3.3%) could not be restored with screw-retained prosthesis. Seven FH implants (23.3%) had fenestration at the apex, and one FH implant (3.3%) suffered from dehiscence. Seven FH implants (23.3%) were not restorable with screw-retained prosthesis.

**Conclusion:**

For novice clinicians, a significantly greater accuracy was observed for FG placement, followed by PG and FH placements. FH implants experienced significant compromise of periimplant bone dimension and the prosthesis retention mechanism.

## Introduction

Ideal implant placement is necessary for successful implant prosthesis from the biological, mechanical, and aesthetic perspectives. 1 2 3 Poorly placed implants are difficult to restore and can cause damage to the adjacent teeth and vital structures. 1 2 4 5 6 7 A higher rate of biological complications, such as soft tissue inflammation and periimplant bone loss, was observed around inadequately placed implants. 2 Placement of implants in prosthetically driven positions can be challenging for implant surgeons with limited clinical experience, and in clinical situations where it is necessary to place implants in a narrow ridge. 8 9 10 11 12 Thus, it is recommended for novice clinicians to execute comprehensive presurgical implant planning and utilize surgical guides to control implant placement. 13

Surgical guides can be conventionally fabricated on diagnostic casts, or digitally designed and fabricated via static computer-assisted implant surgery (sCAIS) workflow. The latter is commonly applied in today's practice due to the availability of numerous three-dimensional (3D) implant planning software, scanning technologies, accessibility of cone-beam computed tomography (CBCT), and the availability of advanced digital fabrication methods such as 3D printing and milling. The advantages of sCAIS were confirmed by several laboratory and clinical studies. This involved the superior accuracy, reduced surgical invasiveness, reduced reliance on bone augmentation graft, and a more predictable aesthetic outcome. 1 4 7 8 14 15 16

sCAIS are available in two forms, pilot-guided (PG) and fully guided (FG) placements. 1 10 The PG placement controls the pilot drill only and the remaining steps are completed freehand (FH), while the FG placement controls all the drilling steps, tapping and the implant placement via precision surgical guide and sleeves. 4 7 14 The PG placement has the advantages of simplicity, use of a standard surgical kit and protocol, and visualization of the drilling procedure and implant placement. 13 On the other hand, the FG placement has been consistently shown to be more accurate than PG placement. 1 4 6 17 18 However, the FG placement mandates the use of a dedicated surgical kit and drills, and a modified surgical protocol to ensure the drills and implants are controlled by the guide. Thus, FG placement has the drawback of complexity and increased cost. 15 19 20 As a result, despite the clear accuracy and benefit of FG placement, earlier studies that argued the benefits of FG placement over PG placement for wide ridges may not be of clinical significance. However, in situations of narrow bone ridge, the clinical benefits of FG placement may become more apparent. For example, in narrow bone ridge, it is more likely for the implant to have dehiscence and fenestration. Placing the implant accurately within the narrow bone ridge will reduce the need and invasiveness of simultaneous bone augmentation. 21 22 Therefore, the aim of this study was to evaluate the accuracy and clinical impact of implant placement by novice implant clinicians in the narrow anterior bone ridge by FG, PG, and FH placements. The clinical impact was evaluated via two variables: (1) periimplant bone dimension and necessity of bone augmentation, and (2) the possibility of screw retention of the definitive prosthesis. These variables were selected because the final implant position and angulation can affect the periimplant bone dimension 2 3 5 and prosthesis retention mechanism. 1 19 This study hypothesizes that, in narrow anterior bone ridges, no differences exist in the accuracy and clinical impact of FG, PG, and FH implant placement protocols by novice clinicians.

## Materials and Methods

Fifteen qualified clinicians (7 males and 8 females) enrolled in postgraduate training involving implant dentistry were requested to participate in the study. All the participants had at least 3 years of experience in general dentistry, but had limited experience with implant dentistry. Prior to the experiment session, the clinicians received theoretical foundation of implant planning and placement. A power calculation (G*Power version 3.1.9.2, University of Dusseldorf, Dusseldorf, Germany) confirmed the number of participants. The sample size calculation was based on the expected effect size of differences among the placement protocols. 4 8 With an alpha level of 5% and a statistical power of 80%, a minimum of 11 participants was required to detect a statistically significant difference.

### Surgical Models and Surgical Guides Fabrication

**Fig. 1:**
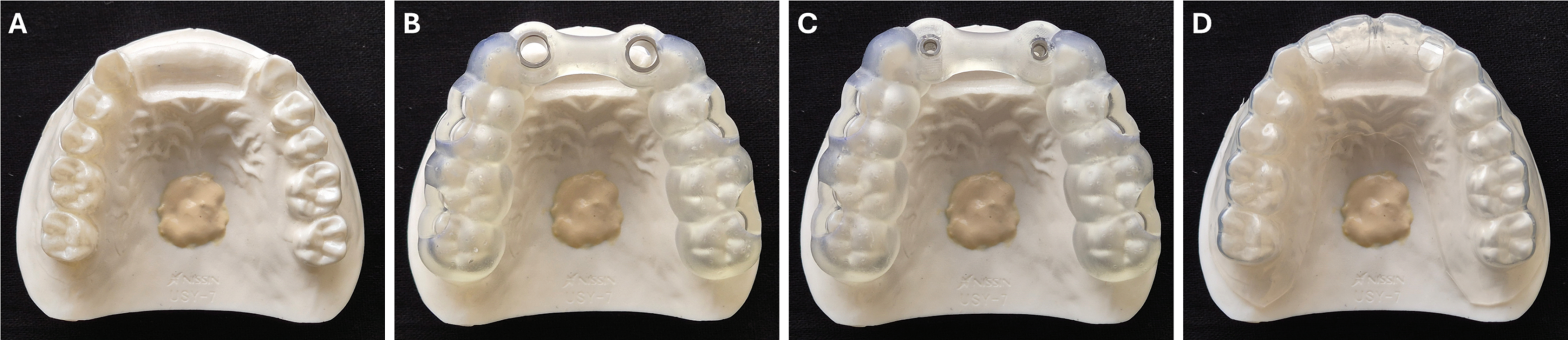
The surgical model used in the study. ( **A** ) Occlusal view of the surgical model used for implant placements at the lateral incisor locations. ( **B** ) A tooth supported surgical guide of the fully guided (FG) placement with the wide metal Straumann sleeves that control all the drills and the implant placement. ( **C** ) An identical surgical guide for the pilot-guided (PG) placement that has narrow metal sleeves to control the pilot drill. ( **D** ) For the freehand (FH) placement, clear thermoplastic surgical guides were produced with perforations on the palatal surfaces of the lateral incisors.

A maxillary training model (Nissin Dental Products Inc., Kyoto, Japan) was modified with the removal of all the incisors and the associated simulated soft tissue. The ridge was further modified to resemble a healed ridge of minimal width (5 mm at the crest with gradual increase to 7 mm at the base of the crest) ( Fig. 1A ). The model was scanned by a laboratory scanner (Identica T300, Medit Identica, DT Technologies, Davenport, Iowa, United States) to generate a virtual model for implant planning and surgical guide designs. Implants were planned for the locations of right and left lateral incisors by a planning software (coDiagnostiX, Dental Wings, Montreal, Canada). The implants were Straumann bone level tapered implants of 4.1 mm diameter and 10 mm length. The planning ensured the implants could be restored by a 4-unit screw-retained implant bridge prosthesis. To facilitate the restorative-driven implant planning, a fully dentate maxillary virtual model was superimposed on the surgical model. The STL file of the virtual model with the planned implants was generated to serve as a master model to quantify the deviations of every placed implant.

Whole arch tooth-supported surgical guides were designed. For the FG placement, the guide was designed to accept metal sleeves of 5 mm diameter at the site of each implant ( Fig. 1B ). The PG guide had an identical design, apart from the incorporation of pilot drilling sleeves of 2.2 mm diameter ( Fig. 1C ). The virtual surgical guides were imported into the 3D printer (Form 3 + , Formlabs, Somerville, Massachusetts, United States) to produce 15 FG and 15 PG surgical guides. Subsequently, the metal sleeves (Straumann AG, Basel, Switzerland) were placed on the corresponding surgical guides. For the FH placement, clear thermoplastic surgical guides were produced on the dentate master model ( Fig. 1D ). The palatal areas were perforated to allow the clinician to drill through the guide and ensure palatal screw access.

The modified model was duplicated by laboratory silicone molding material (Elite Double, Zhermack S.p.A., Badia Polesine, Italy) to produce polyurethane (Easycast, Barnes, Moorebank, Australia) surgical models. A total of three surgical models were produced for each participant. In order to mimic a clinical setup, the surgical models were attached to phantom heads with an opposing fully dentate mandibular model.

### Implant Placements

The implants were inserted initially according to FH placement, followed by PG and FG placements. This sequence was necessary to avoid familiarizing the clinicians of the ideal implant placement with the more restrictive placement techniques. The clinicians had access to the surgical planning images of ideal implant placement. The PG placement controlled the pilot drilling only and the rest of the steps were completed without the guide. The vertical placement of the FH and PG implants was determined by measuring the insertion of each drill within the crest of the model. The FG placement controlled all the drilling steps and implant placement through the guide.

### Accuracy Evaluation

Laboratory scan bodies (ZFX Scan body, ZFX Dental, Zimmer Biomet, Warsaw, Indiana, United States) were attached to the inserted implants and were scanned by the laboratory scanner to generate virtual surgical models. The actual implant positions were determined by matching virtual keys of the scan body and implant. A parametric cylindrical implant body was used to facilitate dimensional measurements. The surgical model with the virtual implants was superimposed on the master model with the planned implants to measure the differences in implant positions by 3D rendering software (Geomagic Control X, Raindrop, Geomagic Inc., Research Triangle Park, North Carolina, United States). The superimposition relied on the teeth of the models. The merged models were used to measure the maximum angle deviation between implant axes, vertical deviation between the centers of implant platforms, and horizontal deviation between the centers of implant platforms and apices. Further, the directions of vertical and horizontal deviations were determined.

### Clinical Impact Evaluation

The clinical impact evaluation aimed to relate the position of implants of each placement protocol to the clinical surgical and prosthetic outcomes. Two clinically relevant variables were considered: (1) periimplant bone dimension after implant placement, and (2) the prosthesis retention mechanism. The two implants were virtually planned to have at least 1.5 mm bone on the buccal aspect and to be restored with screw-retained bridge prosthesis without an angle correction mechanism or cementation. The periimplant bone dimension determined the need of simultaneous bone augmentation following implant placement, where the more favorably placed implant required less simultaneous bone augmentation. This was established by measuring labial bone thickness around the platform and the apex of the implant. The accurately placed implant should be fully covered with bone with at least 1.0 mm of bone at the two locations. The prosthesis retention mechanism of the more favorably placed implant should be screw-retention with access on the palatal surfaces of the lateral incisors. A virtual fully dentate maxillary model was superimposed on each virtual surgical model with the parametric implants. Subsequently, the screw access of each implant was related to the surfaces of the lateral incisors.

### Statistics

For all the variables, the mean and standard deviation (SD) were calculated. The normality of the data was evaluated by the Shapiro–Wilk test, and the one-way analysis of variance test was conducted followed by the Tukey post hoc test. The statistical tests were performed by the SPSS software (SPSS for Windows, version 23, SPSS Inc., Chicago, Illinois, United States), with a 0.05 level of significance. The horizontal mesiodistal and buccolingual platform and apex deviations of the implants were plotted in 3D scatter diagrams.

## Results

### Accuracy

There was a clear tendency for FG implants to exhibit the greatest similarity to the planned implants for all the analyses. The FH placement was noticeably inferior to other forms of placement. Table 1 summarizes the angle, vertical, and horizontal deviations for different implant placements.

**Table 1:** Summary of implant horizontal, vertical, and angle deviations

	Maximum implant angle deviation		
	FG	PG	FH
Mean (degrees)	1.71	4.47	4.62
SD (degrees)	1.08	2.71	3.82
Maximum (degrees)	4.14	12.00	18.60
Minimum (degrees)	0.13	0.50	0.50
*p-* Values	All groups < 0.001 a FG vs. FH = 0.001 b	FG vs. PG = 0.001 b PG vs. FH = 0.98	
	**Magnitude of vertical implant deviation**		
	FG	PG	FH
Mean (mm)	0.57	0.69	0.74
SD (mm)	0.36	0.67	0.64
Maximum (mm)	1.71	2.04	1.92
Minimum (mm)	–0.08	–0.66	–0.56
*p-* Values	All groups = 0.12		
	**Maximum horizontal implant platform deviation**		
	FG	PG	FH
Mean (mm)	0.28	0.43	0.85
SD (mm)	0.18	0.24	0.51
Maximum (mm)	0.77	0.89	2.42
Minimum (mm)	0.05	0.03	0.19
*p-* Values	All groups < 0.001 a FG vs. FH = 0.001 b	FG vs. PG = 0.19 PG vs. FH = 0.001 b	
	**Maximum horizontal implant apex deviation**		
	FG	PG	FH
Mean (mm)	0.55	1.01	1.26
SD (mm)	0.33	0.44	0.64
Maximum (mm)	1.44	2.19	2.49
Minimum (mm)	0.09	0.29	0.51
*p-* Values	All groups < 0.001 a FG vs. FH = 0.001 b	FG vs. PG = 0.001 b PG vs. FH = 0.12	

Abbreviations: ANOVA, analysis of variance; FG, fully guided placement; FH, freehand placement; PG, pilot-guided placement; SD, standard deviation.

a Statistical significant difference according to one-way ANOVA test.

b Statistical significant difference according to Tukey post hoc test.

**Fig. 2:**
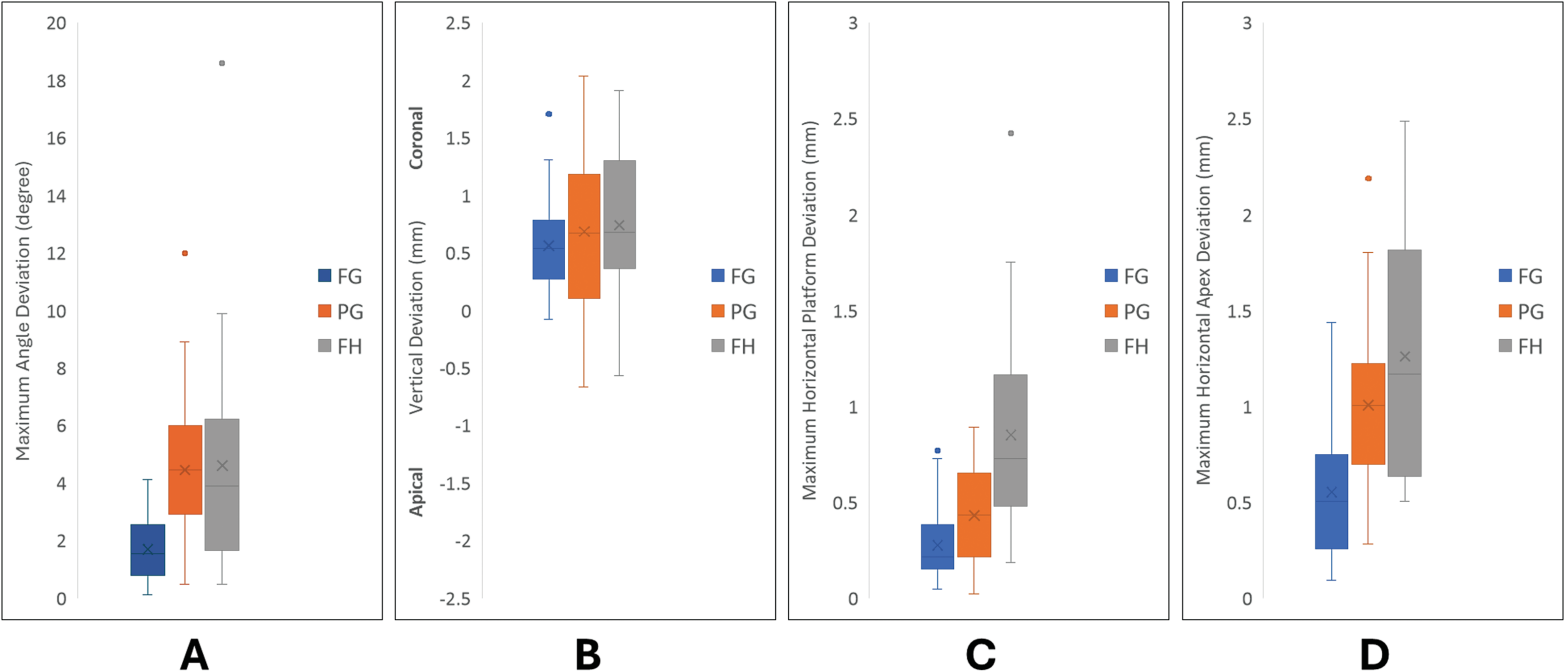
Box-and-whisker plot diagrams illustrating the accuracy at each variable of different placement protocols. ( **A** ) Angle deviation. ( **B** ) Vertical deviation. ( **C** ) Horizontal platform deviation. ( **D** ) Horizontal apex deviation.

For the angle deviation ( Fig. 2A ), the FG implants had the least deviation followed by PG and FH implants ( *p* < 0.001), while the PG and FH implants had relatively similar angle deviations ( *p* = 0.98). In general, the vertical deviation ( Fig. 2B ) was the least for FG implants, followed by PG and FH implants. However, the difference among them was insignificant ( *p* = 0.12). For all placement techniques, there was a tendency for the implants to be located above the planned implant. The FG and PG implants had a similar maximum horizontal deviation at the platform level ( *p* = 0.19) ( Fig. 2C ), and both were more accurate than FH implants ( *p* < 0.001). However, at the apex ( Fig. 2D ), the FG implants were significantly more superior then the other placements ( *p* < 0.001), while the PG and FH placements were similar ( *p* = 0.12). The placed implants showed consistently greater deviation at the apex (approximately twice) than platform for FG ( *p* < 0.001), PG ( *p* < 0.001), and FH ( *p* = 0.008) placements.

**Fig. 3:**
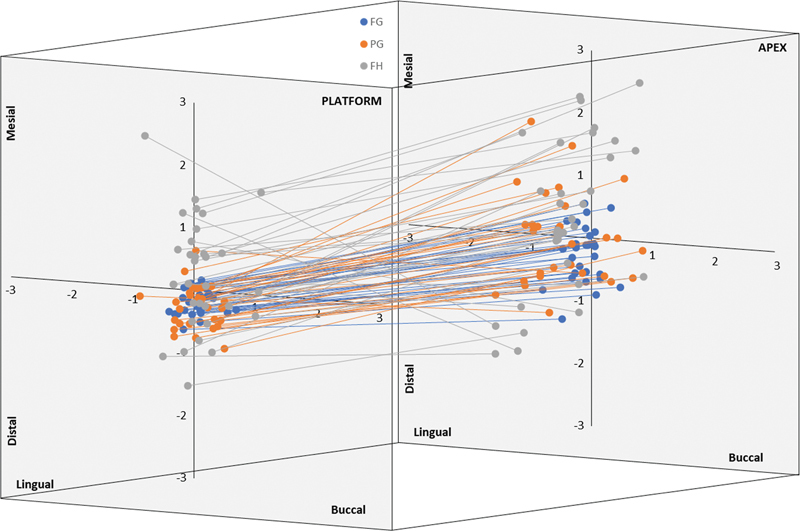
Three-dimensional scatter diagrams illustrating the mesiodistal and buccolingual deviations of the platform and apex of each implant.

The 3D graph ( Fig. 3 ) indicated that the deviations of all implant placements tend to occur more in the mesiodistal direction than the buccolingual direction at the platform and the apex, which could be related to the restricted buccolingual dimension of the ridge. However, the apex showed a clear increase of the buccolingual deviation with greater lingual tendency. The platform and apex of FG implants were mostly centered to the middle of the graph. The apex of the FG implants showed noticeable deviation at the mesiodistal direction. The PG implants had a similar distribution to the FG implants at the platform; however, at the apex, a noticeably wider distribution in all directions was observed at the mesiodistal direction. The FH implants exhibited a wider distribution at the platform, which was further accentuated at the apex. At the platform and apex, the distribution was primarily in the mesiodistal direction.

### Clinical Impact Evaluation

**Fig. 4:**
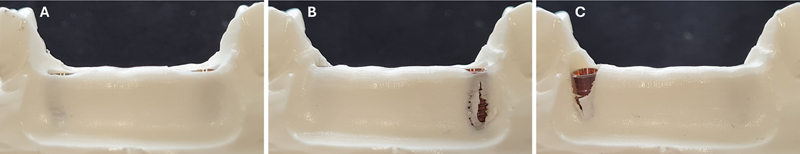
Some examples of the impact of errors associated with freehand (FH) implant placements. ( **A** ) Labial placement of the implants leading to reduced buccal bone thickness. ( **B** ) Fenestration involving most of the implant length. ( **C** ) Dehiscence associated with excessive labial positioning of the implant.

All the FG implants (100%) were fully covered with bone at the platform and the apical portion. One of the PG implants had fenestration at the apical portion (3.3%), while the rest of the implants were fully covered with bone. Seven FH implants had fenestration at the apical portion (23.3%), and one FH implant had dehiscence (3.3%) ( Fig. 4 ). The average buccal bone width around the FG implants was 1.48 mm (SD = 0.33 mm) at the platform, and 1.73 mm (SD = 0.21 mm) at the apex. For the PG implants, the average buccal bone was 1.34 mm (SD = 0.32) at the platform and 1.63 mm (SD = 0.43) at the apex. The FH implants had 0.91 mm (SD = 0.78) buccal bone width at the platform and 1.30 mm (SD = 0.74) at the apex. There was no significant difference between FG and PG implants at the platform ( *p* = 0.49) and apex ( *p* = 0.74); however, both were significantly superior to FH implants ( *p* < 0.05).

**Fig. 5:**
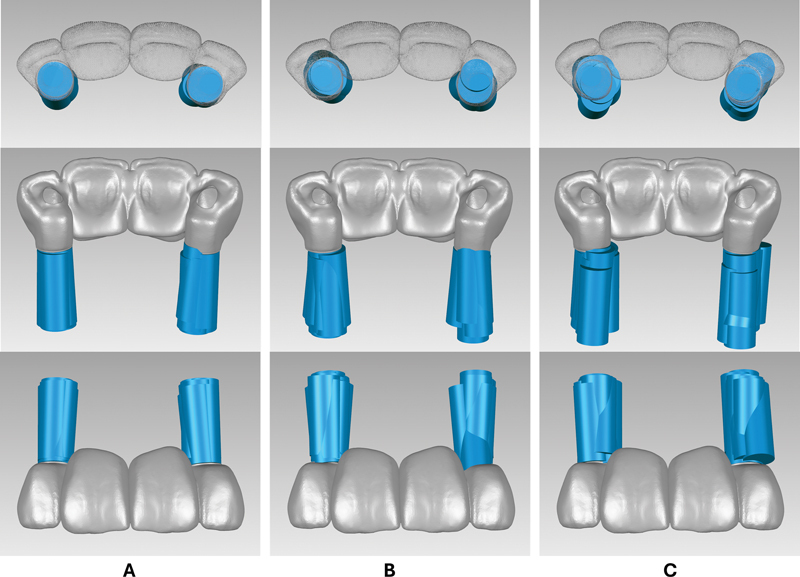
The relationship between the placed implants and the planned screw-retained implant bridge of the different implant placement protocols at the occlusal, palatal, and labial views. ( **A** ) fully guided (FG) placement. ( **B** ) pilot-guided (PG) placement. ( **C** ) freehand (FH) placement.

The screw access analysis revealed that all the FG implants (100%) could be restored by screw-retained prosthesis without angle correction ( Fig. 5A ). One of the PG implants (3.3%) could only be restored with cement retention or an angle correction mechanism due to labial positioning of the implant. Ten PG implants (33.3%) were located palatally, but could still be restored with screw retention ( Fig. 5B ). Four PG (13.3%) implants were tilted mesially which may mandate screw access location at the embrasure area. Seven FH implants (23.3%) could only be restored with cement retention (2 implants had palatal tilt, 4 implants had labial tilt, and 1 implant had mesiopalatal tilt) ( Fig. 5C ). While the remaining FH implants could be restored with screw retention, six implants (20%) had noticeable tilt (3 implants had mesial tilt, 2 implants had palatal tilt, and 1 implant had labial tilt).

## Discussion

The present study indicates that for the narrow anterior ridge, the FG placement was consistently most superior, in terms of accuracy and possible clinical outcome, followed closely by the PG placement in the hands of novice operators. The FH placement clearly had the most inferior accuracy with noticeable clinical implications. The observed advantages of the FG placement further supports the findings of numerous earlier studies. 1 3 4 5 6 9 18 23 24 Therefore, the hypothesis that for a narrow anterior bone ridge, there are no differences in the accuracy and clinical impact of implant placement by the different surgical protocols in the hands of novice implant clinicians was rejected. For narrow ridges with restricted room for implant, it is safer to consider a form of sCAIS, especially if all the steps are fully guided through the surgical guide. Further, the FG placement is likely to reduce the reliance on the bone augmentation procedure, 21 22 and will allow for a more favorable prosthesis retention mechanism. Thus, it can be speculated that for a narrow ridge, the FG placement may reduce the incidence and severity of biological and mechanical complications of implant treatment.

The observed FG placement deviation was similar to previously published reports, where the range of horizontal platform deviation was 0.4 to 1.2 mm, horizontal apex deviation was 0.7 to 1.5 mm, vertical deviation was 0.7 to 1.5 mm, and the angle deviation range was 1.4 to 4.2 degrees. 1 3 4 5 6 18 23 24 The consistently superior accuracy of FG implants over FH and PG implants can be attributed to the control of all the drilling steps and actual implant placement via the surgical guide. 1 3 4 5 6 18 23 24 Interestingly, as the observed FG placement deviations of the present study was similar to earlier studies involving experienced clinicians, 1 4 5 6 9 18 23 it can be speculated that the FG placement removes the disparity of placement errors among clinicians with different levels of experience. 8 9 10 16 25 The susceptibility of FG placement to error has been attributed to the fit of the guide, CBCT and scanning resolution, and the tolerance of the components. 1 3 4 5 6 7 18 23 24 26

This study was associated with unique presentations that may further accentuate the errors of FG and PG placements with the lack of guide support at the anterior segment of the guide above the edentulous area. This could have influenced guide stability during drilling and implant placement. For example, the present study revealed a predominant pattern of FG and PG deviation where the apices tended to deviate lingually. A similar pattern of deviation was observed in earlier investigations where a section of the guide was not supported. 6 17 25 27 28 29 30 31 This direction of error can also be related to the operator placing pressure at the sleeve of the guide, leading to guide distortion and displacement toward the edentulous area leading to the rotation of the implant. 25 31 32 In confirmation of earlier recommendations, a safety zone of 1 to 2 mm should be considered whenever an implant is planned, 4 7 26 an intermittent evaluation of the osteotomy is necessary to avoid significant deviation of FG implants, and excessive pressure on the guide should be avoided. 4 7 23 33

Despite that the accuracy differences between FG and PG implants of the present study were minimal, they were sufficient to affect the clinical outcome of a few PG implants. This can be due to greater errors at the apex and angle for the PG implants compared with the FG implants. The increased errors for PG placement can be attributed to the execution of all the drilling steps apart from the pilot drilling and FH implant placement. While sequential drilling steps are guided by the crestal portion of the pilot osteotomy, the subsequent drill can still move through the pilot osteotomy. This trajectory can lead to greater errors at the apex and angulation of PG implants. 9 10 11 12 According to the present study, the PG placement is still prone to clinically relevant errors, where one PG implant had perforated the ridge apically and another implant could not be restored with simple screw retention. Nevertheless, in line with earlier studies on PG placement, this study confirms the merits of PG placement over FH placement. 9 10 11 12 While the superiority of PG placement over FH placement is only evident at the horizontal platform deviation, FH implants had a significantly inferior clinical outcome (surgically and restoratively) than PG implants, where approximately a quarter of the FH implants suffered from buccal bone perforation, and a quarter of them could not be restored with screw retention. This outcome supports the necessity of using a form of guided implant surgery by novice clinicians whenever the bone ridge is narrow. While bone dehiscence and fenestration can be corrected by simultaneous bone augmentation, the severity of the perforation will influence the invasiveness of the augmentation procedure and its prognosis. 21 22 From this study, it is evident that FH placement in cases of high aesthetic demand and limited bone is associated with an inferior standard of care and significant risks to treatment outcome and patient safety. Thus, the role of routine FH placement should be reevaluated as it can lead to suboptimal outcomes or even malpractice, and a form of guided implant surgery should be the minimal requirements for challenging cases. Wherever the bone volume is limited, and implant placement can affect the periimplant bone dimension and prosthesis design, FG placement should be considered as it is the most accurate form of sCAIS. For example, a clinical study by Younes et al found that the FG implants were more likely to be restored with screw retention as opposed to PG and FH implants. 1 Further, Lou et al found greater reliability of FG implants in achieving a more aesthetic outcome than PG implants after a 1-year follow-up. 3

Laboratory studies on the accuracy of implant placement are limited due to the lack of simulation of clinical challenges such as the presence of blood and saliva, patient movement, variable mouth opening, and variations in ridge anatomy and quality. Several studies indicated that clinical studies tend to show greater deviations than laboratory studies. 7 34 The present study assessed the accuracy of FG placement by a single workflow (software and implant system) and setup, which may exhibit different accuracy to other FG placement workflows, 12 35 or different variables, such as sleeve design and length, implant parameters, and distance from the ridge. 29 36 37 Future studies should be conducted on a larger number of participants with varying levels of implant experience and should include diverse clinical settings. Long-term clinical studies are still needed to evaluate the effect of different implant placement techniques on prosthetic stability, aesthetics, patient satisfaction, and treatment cost-effectiveness, which will further contribute to advancing best practices in implant treatment.

## Conclusion

Within the limitations of this laboratory study, for implant placement in a narrow ridge, a significantly greater accuracy was observed for FG placement, followed by PG and FH placements by novice clinicians. FH implants experienced significant compromise of periimplant bone dimension and the prosthesis retention mechanism. Therefore, it is strongly recommended for novice clinicians to consider a form of guided implant surgery whenever the anatomical structure is restricted.
